# Impact of low HER2 expression on response to CDK4/6 inhibitor treatment in advanced HR + /HER2- breast cancer: a multicenter real-world data analysis

**DOI:** 10.1007/s00404-024-07761-2

**Published:** 2024-10-07

**Authors:** Damian J. Ralser, Verena Kiver, Erich-Franz Solomayer, Caroline Neeb, Jens-Uwe Blohmer, Alina V. Abramian, Nicolai Maass, Florian Schütz, Cornelia Kolberg-Liedtke, Carolin Müller, Anna-Christina Rambow

**Affiliations:** 1 Department of Gynecology and Gynecological Oncology, University Hospital Bonn, University Medical Center Bonn, Venusberg-Campus 1, 53127 Bonn, Germany; 2 Department of Gynecology With Breast Center, Charité – Universitätsmedizin Berlin, corporate member of Freie Universität Berlin and Humboldt-Universität Zu Berlin, Charitéplatz 1, 10117 Berlin, Germany; 3 Department of Gynecology, Obstetrics & Reproductive Medicine, Saarland University Medical Center, Homburg, Saar Germany; 4 Department of Gynecology and Obstetrics, University Medical Center Schleswig-Holstein (UKSH), Campus Kiel, Kiel, Germany; 5 Department of Gynecology and Obstetrics, Diakonissen-Stiftungs-Krankenhaus Speyer, Speyer, Germany; 6 Department of Gynecology and Obstetrics, University Medical Center, Essen, Germany; 7 Department of Anesthesiology, Outcomes Research Consortium, Cleveland Clinic, Cleveland, OH 44195 USA

**Keywords:** HER2 low, CDK4/6 inhibitor, Breast cancer, Trastuzumab deruxtecan

## Abstract

**Purpose:**

CDK4/6 inhibitors (CDK4/6i) represent the first-line therapy approach of choice for patients with hormone receptor-positive, HER2-negative advanced breast cancer (HR + /HER-ABC). Approximately 50% of HR + /HER2-ABC displays low HER2 expression (HER2 low). Recent data emerging from the DESTINY-Breast04 trial demonstrated practice-changing efficacy of the antibody–drug conjugate trastuzumab deruxtecan (T-DXd) in patients with low HER2 expression. Here, we aimed to analyze the impact of low HER2 expression on CDK4/6i therapy response in a well-characterized multicenter HR + /HER-ABC cohort.

**Methods:**

Patients diagnosed with HR + /HER2-ABC who were treated with CDK4/6i in clinical routine between November 2016 and December 2020 at four certified German Breast Cancer Centers were retrospectively identified. The cohort was stratified according to graduation of positivity in HER2 immunohistochemistry (IHC; HER2 zero = IHC score 0 and HER2 low = IHC score 1 + , 2 + /fluorescence in situ hybridization negative). Subgroups were analyzed with regard to progression-free survival (PFS) following CDK4/6i initiation.

**Findings:**

The study cohort comprised *n* = 448 patients. For *n* = 311 patients, HER2 status from the metastatic site was available. *n* = 91 (29.3%) cases were HER2 zero and *n* = 220 cases (70.7%) were HER2 low. There was no significant difference in PFS between the two groups (PFS: 17 months versus 18 months, log-rank *p* = 0.42). Further, we examined the influence of HER2 expression changes between primary and metastatic tissue (*n* = 171; HER2 gain/HER2 loss/HER2 stable expression) on CDK4/6i treatment response. Again, there was no significant difference between these three groups, respectively (PFS: 16 months versus 13 months versus 17 months, log-rank *p* = 0.86).

**Conclusions:**

In our analysis, HER2 status did not have a significant impact on treatment response to CDK4/6i.

## Main

CDK4/6 inhibitors (CDK4/6i) in combination with endocrine therapy (ET) represent the therapeutic mainstay for patients with hormone receptor-positive, HER2-negative advanced breast cancer (HR + /HER2-ABC). Despite recent controversy regarding efficacy differences between the three different approved CDK4/6i, namely Palbociclib, Ribociclib, and Abemaciclib, the overall clinical benefit of CDK4/6i is uncontroversial as demonstrated in several randomized clinical trials and published real-world data [1–5].

However, patients with HR + /HER-ABC comprise a heterogenous population and a substantial patient proportion show disease progression on first-line CDK4/6i-based therapy. Beyond different histopathological subtypes, estrogen and progesterone receptor expression, histomorphological grading and Ki-67 index, the HER2 receptor exhibits varying expression levels across HR + /HER2-ABC. Research has shown, that approximately 50% of HR + /HER2-ABC displays low HER2 expression designated by graduation of positivity in HER2 immunohistochemistry (IHC; HER2 zero = IHC score 0 and HER2 low = IHC score 1 + , 2 + /fluorescence in situ hybridization negative) [6–9]. This is of the highest clinical relevance as data from the DESTINY-Breast04 trial demonstrated practice-changing efficacy of the antibody–drug conjugate (ADC) trastuzumab deruxtecan (T-DXd) in patients with low HER2 expression [10]. Further, recent data from the DESTINY-Breast06 trial showed a statistically significant and clinically meaningful PFS benefit for T-DXd compared to treatment of physicians choice in patients who experienced disease progression on ET for HR + /HER2-ABC (NCT04494425;[11, 12]). Of note, this applies for both the group of patients with HER2 low and for patients with HER2 ultralow (IHC score 0 with membrane staining).

To date, few data exist to which extent HER2 expression impacts response to CDK4/6i. In the present study, we aimed to analyze the impact of HER2 expression on CDK4/6i therapy response in a well-characterized multicenter HR + /HER-ABC real-world data cohort.

Patients diagnosed with HR + /HER2-ABC who were treated with CDK4/6i in clinical routine between November 2016 and December 2020 at four certified German Breast Cancer Centers (Saarland University Medical Center, University Medical Center Charité Berlin, University Medical Center Bonn and University Medical Center Schleswig–Holstein, Campus Kiel) were retrospectively identified. A recent follow-up was conducted in January 2023.

The cohort was stratified according to graduation of positivity in HER2 immunohistochemistry (IHC; HER2 zero = IHC score 0 and HER2 low = IHC score 1 + , 2 + /fluorescence in situ hybridization negative). Subgroups were analyzed with regard to progression-free survival (PFS) following CDK4/6i initiation. Statistical analyses were performed using SPSS 28.0 (IBM, Armonk, USA) and GraphPad Prism software (GraphPad software, San Diego, CA, USA). Kaplan–Meier analysis and Cox regression were performed to analyze PFS and confounders on PFS.

**Fig. 1:**
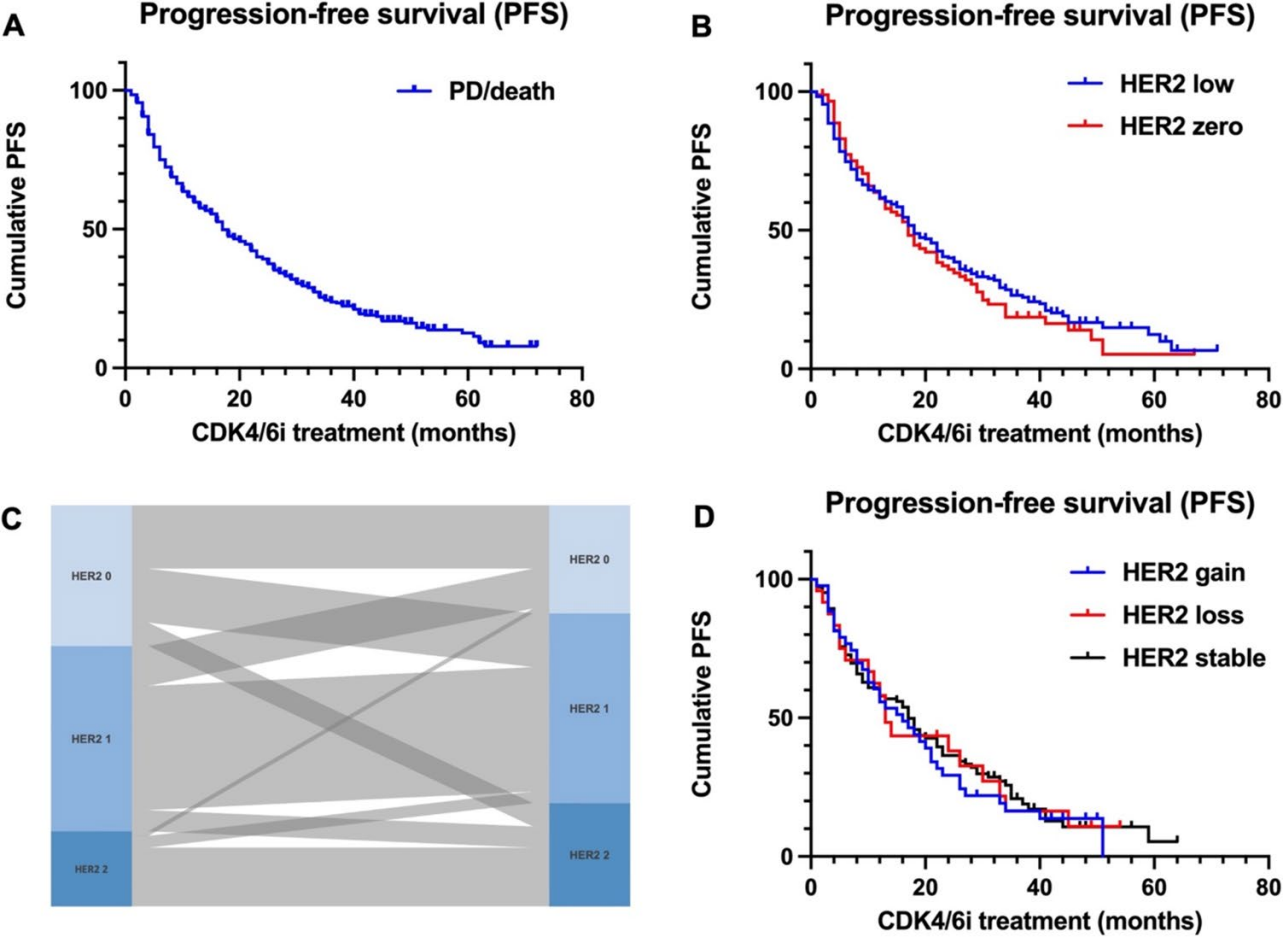
**A** Progression-free survival in Kaplan–Meier analysis following CDK4/6 inhibitor treatment in the entire study cohort (median PFS 17 months). **B** Median PFS depending on HER2 expression (metastatic site). Patients with HER2 zero had 17 months median PFS, patients with HER2 low (IHC score 1 + and 2 +) had 18 months median PFS. Log-Rank *p* = 0.42. **C** Sankey diagram illustrating HER2 expression changes. In *n* = 171 patients, HER2 receptor expression was available for both, primary and metastatic tissue. *n* = 42 patients (24.5%) had HER2 receptor gain, *n* = 24 (14.1%) patients had loss of HER2 expression, and *n* = 105 patients (61.4%) displayed constant HER2 expression. **D** PFS in Kaplan–Meier analysis following CDK4/6 inhibitor treatment showed no difference between patients with HER2 receptor gain/loss/stable expression (16 months versus 13 months versus 17 months, log-rank *p* = 0.86)

A detailed characterization of the study cohort has been published previously [5]. In total, *n* = 448 patients were included in the final analysis. *n* = 319 patients (71.3%) received Palbociclib, *n* = 114 patients (25.4%) received Ribociclib, and *n* = 15 patients (3.3%) received Abemaciclib. *n* = 165 (36.8%) patients exhibited primary metastatic breast cancer, and *n* = 283 (63.2%) patients had secondary metastatic disease (Table 1). HER2 expression status from metastatic tissue was available for *n* = 311 cases (69.4%). *n* = 91 (29.3%) cases were HER2 zero, *n* = 154 cases (49.5%) were HER2 1 + , and *n* = 66 cases (21.2%) were HER2 2 + / fluorescence in situ hybridization (FISH) negative (Table 1). The median progression-free survival (PFS) following CDK4/6i therapy initiation was 17 months in the entire population (Fig. 1A). The cohort was stratified according to graduation of positivity in HER2 IHC in HER2 zero (IHC score 0) and HER2 low (IHC score 1 + , 2 + /FISH negative). Examining the median PFS in relation to the HER2 status in metastatic tissue revealed no significant difference. The median PFS was 17 months for patients with HER2 zero and 18 months for patients with HER2 low status, respectively. The differences between the groups were not statistically significant (Fig. 1B; HER2 0 vs. HER2 low (Log-Rank *p* = 0.42)). Of note, the PFS following CDK4/6i therapy initiation in the study cohort is shorter compared to the pivotal trials. This is attributable to the real-world population and in particular CDK4/6i treatment in later lines of therapy (Table 1**,** [5]). Furthermore, we examined the influence of HER2 expression changes during metastatic evolution on PFS. For this purpose, patients who had secondary metastatic disease were divided into (i) patients with gained HER2 expression (initial HER2 zero and in metastatic tissue HER2 1 + or 2 + /FISH-negative; or initial HER2 1 + and in metastatic tissue HER2 2 + /FISH-negative), (ii) patients with constant HER2 receptor expression and (iii) patients with loss of HER2 expression. In *n* = 171 patients, HER2 receptor expression was present in both, primary and metastatic tissue. In this subgroup, *n* = 42 patients (24.5%) had HER2 receptor gain, *n* = 24 (14.1%) patients had loss of HER2 expression, and *n* = 105 patients (61.4%) displayed constant HER2 expression (Table 2**, **Fig. 1C). No PFS difference was observed between these three subgroups (16 months versus 13 months versus 17 months, log-rank *p* = 0.86; Fig. 1D).

**Table 1:** Cohort characteristics

	(*n*)	(%)
Primary metastatic disease	165	36.8
Secondary metastatic disease CDK4/6i Palbociclib Ribociclib Abemaciclib	283 319 114 15	63.2 71.3 25.4 3.3
Disease site		
Osseous	334	74.6
Hepatic	133	29.7
Pulmonary	154	34.4
Other (lymphatic, peritoneal, cerebral)	219	48.9
Therapy line		
1	278	62.1
2	86	19.2
> 3	84	18.7
HER2 receptor status (metastatic disease)	311	
Her2 0	91	29.3
Her2 1 +	154	49.5
Her2 2 +	66	21.2

**Table 2:** HER2 receptor switch

HER2 status Primary Tumor	(*n*)	(%)	HER2 status Metastatic Tumor	(*n*)	(%)
HER2 IHC 0	60	35.1	HER2 IHC 0	27	45.0
			HER2 IHC 1 +	23	38.3
			HER2 IHC 2 +	10	16.7
HER2 IHC 1 +	79	46.2	HER2 IHC 0	17	21.5
			HER2 IHC 1 +	53	67.1
			HER2 IHC 2 +	9	11.4
HER2 IHC 2 +	32	18.7	HER2 IHC 0	2	6.3
			HER2 IHC 1 +	5	15.6
			HER2 IHC 2 +	25	78.1

Data are presented for patients with secondary metastatic disease in which HER2 receptor expression data was available from primary breast cancer tissue (*n* = 171). HER2 receptor switch is illustrated in Fig. 1C

These data are in line with recently published data that demonstrated that approximately 38% of patients display changes of HER2 expression between primary tumor and metastasis [9, 13–15].

With regard to ET response, experimental research has shown that HER2 expression promotes ET resistance [16–18]. However, clinical attempts to restore endocrine sensitivity by combined endocrine and HER2-directed therapy failed [19, 20]. Experimental data showed that CDK4/6i treatment response was independent of HER2 expression [21]. Similarly, in the clinical context of the current first-line regimen, HER2 status appears to exert no influence on treatment response, as demonstrated in the current study. In contrast to that, a recently published retrospective, multicentric observational study from Italy involving *n* = 428 patients demonstrated that HER2 low status was associated with worse PFS compared to HER2 zero (median PFS 23.6 months vs. 32.3 months; *p* = 0.014) [22]. However, only patients treated with first-line ET + /CDK4/6i were enrolled [22].

Ongoing scientific efforts aim to identify biomarkers to identify patients who do not benefit from CDK4/6i-based therapy. Potential contemporary alternative regimens in this treatment setting include conventional chemotherapy and ADCs e.g., T-DXd and Sacituzumab govitecan, both of which have been approved by the European Medicines Agency (EMA) for treatment of HR + /HER2-ABC after disease progression on ET and one to two lines of systemic therapy lines, respectively. Recent data from the DESTINY-Breast04 and DESTINY-Breast06 trials strongly encourage a consideration of this substance in the therapy algorithm of HR + /HER2-ABC [10–12].

To date, the use of the intensity of HER2 receptor expression as a potential predictive biomarker remains uncertain. Although the biological mechanism of action of ADC suggests a relationship between target receptor expression and treatment response, data on different ADCs demonstrated varying results [23, 24].

We acknowledge the limitation of our analysis (i.e., retrospective nature). Discrepancies among studies in HER2 low patients might be attributed to varying methods of HER2 status assessment, differences in patient populations (such as distinct patient and tumor characteristics), and diverse clinical management approaches (including different types of CDK4/6i, backbone endocrine therapy or differing subsequent lines of therapy).

However, in our multi-center HR + /HER2-ABC cohort, HER2 expression status had no impact on response to CDK4/6i therapy. Our data are consistent with previously published studies in which HER2 expression status also had no prognostic value on CDK4/6i therapy response [10, 11]. Therefore, HER2 expression status might not be a biomarker that defines a subset of HR + /HER2-ABC in terms of therapy response to CDK4/6i. Further studies are needed in this regard.

## Data Availability

The datasets generated and/or analyzed during the current study are available on request from the authors.

## Abbreviations

CDK4/6i: CDK4/6 inhibitors
HR + /HER-ABC: Hormone receptor-positive, HER2-negative advanced breast cancer
T-DXd: Trastuzumab deruxtecan
IHC: Immunohistochemistry
PFS: Progression-free survival
ADC: Antibody–drug conjugate
ET: Endocrine therapy
EMA: European medicines agency
